# Accurate implant fit and leg alignment after cruciate-retaining patient-specific total knee arthroplasty

**DOI:** 10.1186/s12891-020-03707-2

**Published:** 2020-10-22

**Authors:** Jörg Arnholdt, Yama Kamawal, Konstantin Horas, Boris M. Holzapfel, Fabian Gilbert, Axel Ripp, Maximilian Rudert, Andre F. Steinert

**Affiliations:** 1 Department of Orthopaedic Surgery, König-Ludwig-Haus, Julius-Maximilians-University Würzburg, Brettreichstraße 11, D-97074 Würzburg, Germany; 2 Regenerative Medicine, Institute of Health and Biomedical Innovation, Queensland University of Technology, 60 Musk Ave, Kelvin Grove, Brisbane, 4059 Australia; 3 Department of Trauma, Hand, Plastic and Reconstructive Surgery, Julius-Maximilians-University Würzburg, D-97080 Würzburg, Germany; 4 Department of Trauma and Orthopaedic Surgery, Elblandklinikum Radebeul, Heinrich-Zille-Straße 13, D-01445 Radebeul, Germany; 5 Rhön-Klinikum Campus Bad Neustadt, Department of Orthopaedic, Trauma, Shoulder and Arthroplasty Surgery, Von-Guttenberg-Straße 11, D-97616 Bad Neustadt a. d. Saale, Germany

**Keywords:** Total knee replacement, Knee axis, Patient-specific knee arthroplasty, Knee osteoarthritis, Implant positioning

## Abstract

**Background:**

For improved outcomes in total knee arthroplasty (TKA) correct implant fitting and positioning are crucial. In order to facilitate a best possible implant fitting and positioning patient-specific systems have been developed. However, whether or not these systems allow for better implant fitting and positioning has yet to be elucidated. For this reason, the aim was to analyse the novel patient-specific cruciate retaining knee replacement system iTotal™ CR G2 that utilizes custom-made implants and instruments for its ability to facilitate accurate implant fitting and positioning including correction of the hip-knee-ankle angle (HKA).

**Methods:**

We assessed radiographic results of 106 patients who were treated with the second generation of a patient-specific cruciate retaining knee arthroplasty using iTotal™ CR G2 (ConforMIS Inc.) for tricompartmental knee osteoarthritis (OA) using custom-made implants and instruments. The implant fit and positioning as well as the correction of the mechanical axis (hip-knee-ankle angle, HKA) and restoration of the joint line were determined using pre- and postoperative radiographic analyses.

**Results:**

On average, HKA was corrected from 174.4° ± 4.6° preoperatively to 178.8° ± 2.2° postoperatively and the coronal femoro-tibial angle was adjusted on average 4.4°. The measured preoperative tibial slope was 5.3° ± 2.2° (mean +/− SD) and the average postoperative tibial slope was 4.7° ± 1.1° on lateral views. The joint line was well preserved with an average modified Insall-Salvati index of 1.66 ± 0.16 pre- and 1.67 ± 0.16 postoperatively. The overall accuracy of fit of implant components was decent with a measured medial overhang of more than 1 mm (1.33 mm ± 0.32 mm) in 4 cases only. Further, a lateral overhang of more than 1 mm (1.8 mm ± 0.63) (measured in the anterior-posterior radiographs) was observed in 11 cases, with none of the 106 patients showing femoral notching.

**Conclusion:**

The patient-specific iTotal™ CR G2 total knee replacement system facilitated a proper fitting and positioning of the implant components. Moreover, a good restoration of the leg axis towards neutral alignment was achieved as planned. Nonetheless, further clinical follow-up studies are necessary to validate our findings and to determine the long-term impact of using this patient- specific system.

## Background

Although many advances in TKA were made in the last decades recent studies showed that still around 19% of the patients treated with total knee replacement suffer from discomfort in their treated knee [1]. The correct fit and positioning of the implants have been identified as crucial parameters for the functional outcome after TKA surgery [2]. Concomitantly, it is well known that malalignment or instability by malpositioning of the components can lead to a higher risk of implant failure and poorer outcome with a higher revision rate over time [3, 4]. For example, Hadi et al. reported the effect of malalignment on revision rates to be modest [5]. To overcome these issues patient-specific knee replacements have been developed. At first, partial knee arthroplasties [6–9] and afterwards total knee replacements have been introduced. These new implants are designed to enable a perfect coverage of the bony surfaces of the tibia and femur and thus, allowing for the patients’ normal knee kinematics. The patient-specific cruciate-retaining knee replacement system iTotal™ CR G2 provides custom-made implants and instruments and is a novel technique for the therapy of patients with tricompartmental gonarthrosis. This system uses computed tomography (CT) scans and a computer-aided design and manufacturing (CAD/CAM) technology to facilitate an ideal fit of the prosthesis components and instruments [10]. Excellent postoperative radiological and few clinical findings using this new technology for uni- [6, 7, 11] (UKA) and bicompartimental [8, 9] knee arthroplasty (BKA) have already been published reporting promising results. The goal of this retrospective analysis is to postoperatively assess the fit and the positioning of the implants. We hypothesized that the treatment with iTotal™ would allow for a correction of the HKA towards an appropriate alignment of 180° and a proper implant fit.

## Methods

### Patients

This retrospective study was conducted in accordance with the Declaration of Helsinki and approved by the institutional review board (IRB) of the Julius-Maximilians-University Würzburg (Nr.: 2016101401). In total, 106 patients (62 women and 44 men) were all treated by using a customized patient-specific cruciate-retaining knee resurfacing system (iTotal™ CR G2; ConforMIS, Inc.; Burlington, MA, USA) from 2011 until 2013. Of these, 47 were right knee implants and 59 left implants. In 51 of the patients patellar resurfacing was conducted. The product has a CE marking and is approved by the United States Food and Drug Administration (FDA). At time of the surgery the average age of the patients was 62.8 ± 6.63 years (range 48–77 years). Patients presenting with a deformity of greater than 15° valgus, varus or flexion or an instability of the ligaments were excluded from the study.

### Pre-operative planning

A computed–tomography scan of the affected leg was routinely conducted preoperatively by scanning the knee, the femoral head and talus center in accordance with a standard protocol (http://www.conformis.com/healthcare-professionals/imaging-professionals) as previously described [10].

**Fig. 1:**
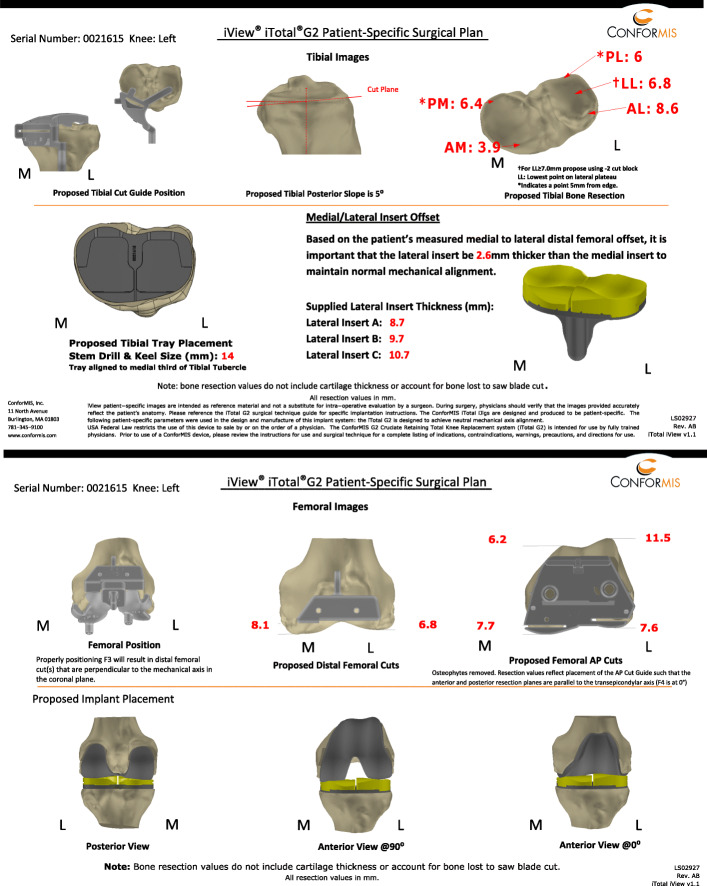
Representative surgical plan (iView™ 2.0) for a cruciate-retaining tricompartmental knee replacement (With kind permission from ConforMIS Inc. as the holder of the copyright of this image)

This customized patient-specific TKA system is designed using a software algorithm (iFit™- Technology) that records the articular surfaces of the knee, the formation of osteophytes and the alignment of the leg. According to this data a TKA is designed which perfectly fits to the anatomy of the patient and further correction of any malalignment is calculated. The patient-specific implants and instruments are then manufactured using 3D CAD/CAM technology. Additionally, an illustrative surgical planning protocol (iView™ 2.0) (Fig. 1) comprising six tibial (upper panels) and six femoral (lower panels) planning images is being prepared. The tibial images show the planned placing of the tibial cutting guide, the planned tibial slope with the proposed height of the resection for the tibial plateau, and the positioning of the tibial component as well as the insert height options (Fig. 1; upper panels). The femoral images provide data on the positioning of the cutting jigs for the distal femur and the anterior and posterior femoral condyles. Furthermore, images of the final proposed implant positioning from a posterior and anterior view in 0° and 90° of flexion are presented (Fig. 1; lower panels). Patient-specific jigs are designed to comprise all crucial informations on the designated geometry, mechanical and anatomical axes of the patients knee joint, as well as the planned cutting planes. The femoral and tibial components are designed in such a way as to enable an exact rim coverage of the cortical bone stock and to the articular surface (Fig. 1), a neutral mechanical alignment and a restoration of the femoral J-curve as described previously [10].

### Surgical technique

A midline skin incision was performed followed by a medial parapatellar arthrotomy. Full exposure of the knee joint was achieved to confirm the indication of tricompartmental knee osteoarthritis with a preserved posterior-cruciate ligament. Next, menisci and any remnants of the anterior-cruciate ligament were excised along with osteophytes interfering with the cutting jigs. The femoral cartilage was then removed using the F1 jig and a core drill followed by the distal femoral resection using the patient-specific cutting blocks F2 and F3. Removed bony pieces were then removed and compared with those displayed in the iView. Afterwards, the tibial cartilage was removed and the tibial resection performed using a specific tibial cutting jig for the anatomic slope of 5° with an attached extramedullary guide to check the axial alignment. Balancing the knee in extension was then performed using individually designed balancing chips and straight axial alignment was confirmed. In general, a proximal tibia cut was performed according to the “zero cut” of the system. However, in few cases further resection was necessary to achieve an appropriate knee balancing. Thereafter, balancing of the flexion gap was achieved by using a patient-specific spacer block in 90° of flexion and removal of all osteophytes interfering with the cuttings jigs. The final preparation of the femur was facilitated by a gap balanced placement of the femoral cutting jigs for the anterior, posterior and chamfer cuts. After kinematic testing using anatomic trial components, the most appropriate insert heights of the medial and lateral knee joint space were identified and the final tibial preparation was performed, again using an individual drill jig that facilitates the correct rotation position of the tibial plateau. The final implant components were then cemented in extension followed by removal of excess cement before the final inlays were inserted. Then wound layers were closed by sutures. Placing a drainage was optional and applied if required. Moreover, patella resurfacing was conducted if necessary, using a standard oval dome patella resurfacement. Collectively, all cutting steps were correlated to the morphology and height of the cutted bone pieces as provided by the iView™. Notably, the detailed surgical procedure has been described previously [10].

### Radiographic analysis

**Fig. 2:**
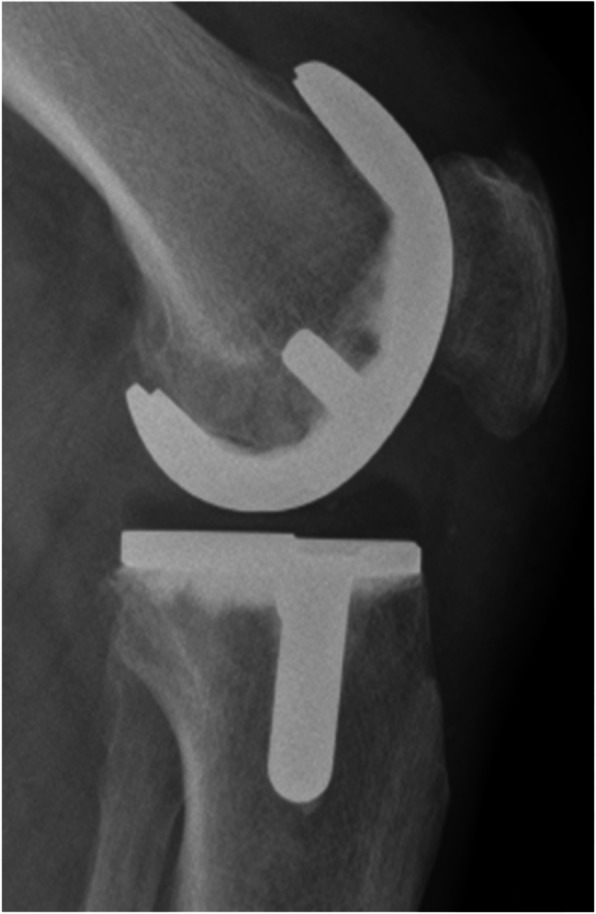
Lateral postoperative radiographic view of a representative knee joint after iTotal™ CR G2 implantation

**Fig. 3:**
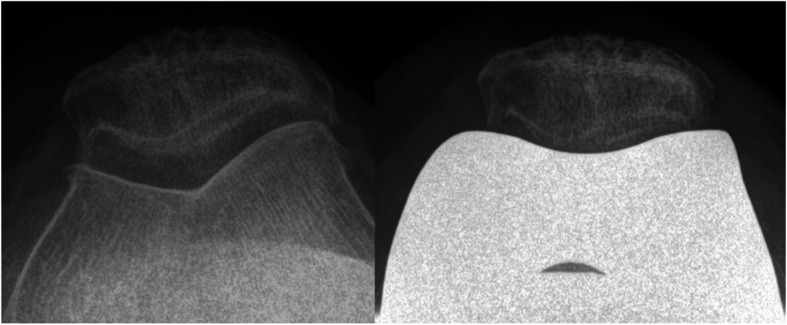
Skyline view of the knee joint before and after iTotal™ CR G2 implantation

**Fig. 4:**
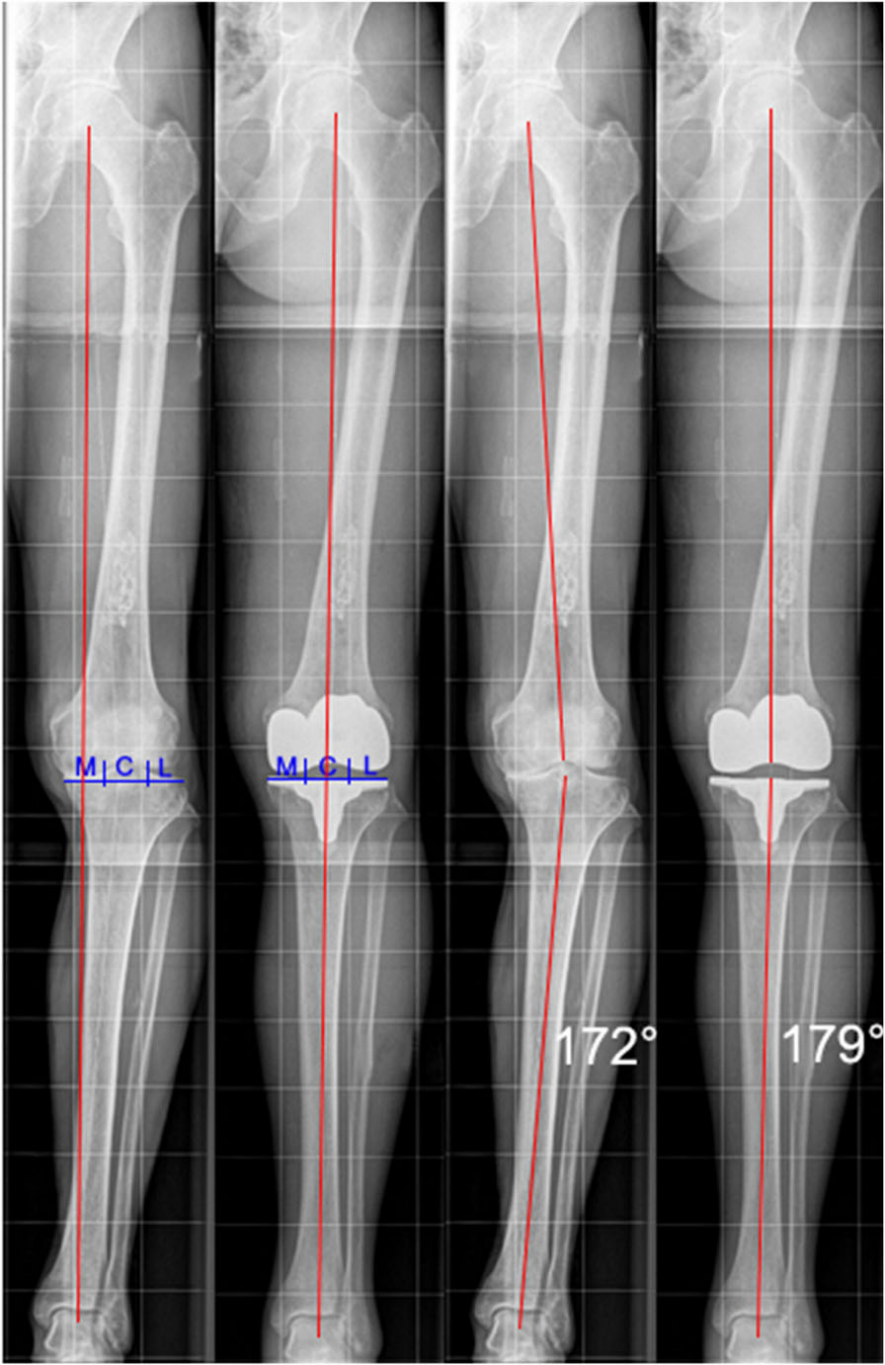
The angle between the mechanical axis of the femur and the mechanical axis of the tibia (red lines) was determined as hip-knee-ankle angle (HKA), and measured in this case 172° preoperatively (**a**), and 179° postoperatively (**b**). To determine the zone of the mechanical axis, the tibial plate was divided into three equal zones (lateral = L, central = C, medial = M) and the mechanical axis (red line) that passes the tibial plate was defined according to which zone is crossed. In this case, medial preoperatively (**c**) and central postoperatively (**d**)

Radiographic evaluations were performed prior to surgery and 1 week post-surgery using a strict antero-posterior (AP) view, a lateral view (including a referencing sphere) as well as a skyline view. Furthermore, AP standing long leg radiographs in the coronal plane were conducted in full extension. The fit of the tibial component was assesed on postoperative AP views. A deviation of > = 1 mm overhang was considered as abnormal. On preoperative lateral views and using the iView™ planning protocol (Fig. 1) the tibial slope was measured and compared to the values measured on postoperative lateral views. As indicated by the iView™ planning protocol one goal of the procedure is to restore the patient-specific anatomic tibial slope. Further, lateral radiologic views were used to detect any variation of the patella height pre- and postoperatively. As a sign for alterations of the joint line (Fig. 2) the Insall-Salvati ratio and the modified Insall-Salvati ratio were measured and calculated [12]. The lateral patella tilt was quantified by analysis of skyline views [13, 14] (Fig. 3). Weight bearing, full-leg radiographs were conducted in order to measure any deviation from the desired mechanical axis. As regards, we determined the angle between the mechanical axis of the femur (FMA) and the mechanical axis of the tibia (TMA) - the so called hip-knee-ankle angle (HKA) (Fig. 4). The TMA is the connection between the center of the ankle and the knee, whereas the FMA connects the center of the femoral head with the center of the knee. 180° ± 3° varus/valgus was considered as the ideal HKA. Importantly, all measurements and calculations were performed by two independent reviewers and all measurements were conducted twice (orthopedic surgeons; JA, YK). Means of all measurements were then used for further comparison.

### Statistical analysis

Microsoft Excel 2007 (Microsoft Corporation, Redmond, USA) was used for descriptive analyses (mean ± standard deviation). Normal distribution (Gaussian distribution) of the collected data was confirmed using the Kolmogorov–Smirnov test. A paired t-test using SPSS (IBM, Germany) was used to compare values of means for determined parameters pre- and postoperatively. Level of statistical significance was set at *p* < 0.05.

## Results

### Implant positioning

Overall, 91 patients presented with a proper implant fit of the tibial component, i.e. an overhang of less than 1 mm on the AP radiographs. A minor overhang (> = 1 mm) on the medial side of the tibial component was recorded in 4 cases (1.33 mm ± 0.32 mm) and in 11 patients (1.8 mm ± 0.63 mm) on the lateral side. None of the cases had an overhang/underhang of 3 mm or more in any of the planes measured. A representative lateral radiographic view presenting an ideal fit of the femoral and tibial implant components without any signs of significant over-, or underhang, or notching is shown in Fig. 2.

The slope of the tibial component was constructed to preserve the natural slope of the tibial plateau. On lateral views of x-rays the mean tibial slope preoperatively was measured with 5.3° ± 2.2°, and 5° in the planned iView protocol. Postoperatively, the tibial slope was reproduced to a mean slope of the tibial implant of 4.7° ± 1.1° upon determination on postoperative lateral view x-rays (Fig. 2).

As demonstrated by the values of the modified Insall-Salvati ratio the patella height was restored. The average modified Insall-Salvati ratio was 1.66 ± 0.16 preoperatively and 1.67 ± 0.16 postoperatively. According to this we could not detect any relevant deviation for the Insall-Salvati ratio measuring 1.02 ± 0.12 preoperatively and 1.03 ± 0.12 postoperatively.

Moreover, evaluation of skyline view radiographs revealed a central patella tracking in all cases postoperatively, without any pathologic lateral patella tilt in any of the cases (Fig. 3).

The thickness of the osseous pieces removed after the distal, anterior and posterior femoral condyle cuts corresponded appropriately to the preoperatively projected resection heights provided by the respective iView™ planning protocol (Fig. 1). The bony cuts of the resected distal, anterior and posterior condyles exactly matched the iView plans in all the cases, which reaffirmed the precision of the patient-specific jigs.

### Frontal plane alignment

**Fig. 5:**
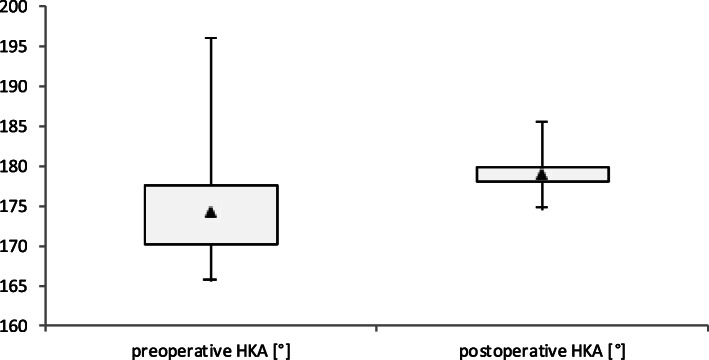
Box plot of the range of the hip-knee-ankle angle (HKA) preoperative and postoperative with illustration of the median angle (**▲**), the area of 25–75% of the cases (**□**), as well as the maximum (**┬**) and minimum scores (**┴**)

The HKA as measured in the radiographic analysis was corrected in all 106 patients from 174.4° ± 4.6° preoperatively to 178.8° ± 2.2° postoperatively (Figs. 4 and 5). The maximum and minimum values of the pre- and postoperative HKA of the study population, and the 25–75% interval are shown in Fig. 5. The tibial plateau was divided into three equal zones (lateral = L, central = C, medial = M). The mechanical axis that passes the tibial plateau was defined according to which zone it crosses and as such defining the zone of the mechanical axis (ZMA) (Fig. 4). After implantation of the iTotal™ CR G2 knee resurfacing system the ZMA improved from 28.3% of cases preoperatively crossing within the central third of the tibial plateau to over 87.7% of cases postoperatively (Table 1).

**Table 1:** Preoperative and postoperative mean HKA (hip-knee-ankle angle) after iTotal™ CR G2 implantation (*n* = 106); outliers (3°/5°) beyond 180° alignment; percentage of ZMA crossing the central third

	Mean ± SD	Outlier ±3°	Outlier ±5°	ZMA central
HKA preoperative	174.4° ± 4.6°	75.5%	60.4%	28.3%
HKA postoperative	178.8° ± 2.2°	18.9%	4.7%	87.7%

As no strong valgus or varus knees were included in the study, we did not encounter any intra-operative difficulties. Furthermore, all patient-specific cutting blocks, drill guides and implants matched precisely to the patients anatomy of the knee joint, so that no intraoperative system change or a modification of the patient specific instruments were necessary.

## Discussion

The patient-specific iTotal™ CR G2 total knee replacement system facilitated a proper fitting and positioning of the implant components. Moreover, a good restoration of the leg axis towards neutral alignment was achieved as planned.

In general, patients that underwent either THA or TKA report of a quick pain relief and a functional improvement. Nonetheless, there are still a number of patients that suffer from postoperative pain and persistent limited range of movement [1, 15] In order to improve the overall outcome of patients novel PSI have been developed. Gong et al. have recently demonstrated that patient-specific instrumentation is associated with improved axial alignment of the femoral component, operative time and perioperative blood loss after TKA [16]. Further, Leon-Munoz et al. have reported that patient-specific instrumentation might reduce operative time, could reduce perioperative blood loss and provides logistical benefits in the operation room [17]. However, no significant differences were found between patient-specific instrumentation and standard instrumentation with respect to alignment of the remaining components, number of outliers or length of hospital stay [16]. Nonetheless, most of the available studies focus on patient-specific instruments rather than on instruments and the patient-specific implants. For this reason, clinical studies focusing on PSI are required to determine whether or not the outcome of patients is better compared to “of the shelf” implants. For this reason, this study intended to analyse the novel patient-specific cruciate retaining knee replacement system iTotal™ CR G2 that utilizes custom-made implants and instruments for its ability to facilitate accurate implant fitting and positioning including correction of the HKA.

This study demonstrates that using the presented system of patient-specific cutting-guides and implants for TKA it was able to appropriately facilitate a correction of the mechanical axis toward neutral leg alignment and an adequate positioning of the femoral and tibial implant components. Notably no substantial overhang upon investigation of frontal plane alignment and lateral views was observed (Figs. 4 and 5).

In this regard, studies on patients with unicompartmental medial knee arthroplasty have shown that an implant overhang of more than 2 mm are potential causes for persisting knee pain [18]. Moreover, the risk for increased pain level due to medial overhang has also been reported in TKA [19]. For this reason, optimal implant position is considered to be pivotal to avoid persistent postoperative pain.

The implant can therefore be placed in ideal position without causing risks due to tibial overhang and following increased pain level.

Along with improved implant positioning correction of leg alignment in the frontal plane in TKA has also been demonstrated to be an important factor. Generally leg alignment outside 3° of varus/valgus of the mechanical axis has been associated with reduced survivorship of TKA [20] and is therefore of high importance when performing TKA. In conventional TKA, correction of the leg alignment is achieved by using intramedullary or extramedullary alignment guides. These are known to be susceptible to substantial errors due to faulty planning, anatomic variability of medullary canals, incorrect entry points, femoral and/or tibial bowings and variability or excessive soft-tissue coverage on external landmarks such as the tibial tubercle or the anterior tibial cortex. Cuts using intramedullary guides have been shown to be faulty with up to 8° [21]. Other authors have reported, up to 8,5% of cuts being suboptimal using conventional techniques [22]. Several other studies have demonstrated that factors such as the optimal surgical techniques [22, 23], the use of computer navigation [24], or patient-specific instruments [25, 26] are able to improve the outcome of patients. Especially the use of computer navigation and the use of PSI are thought to overcome the above mentioned accuracy limitations at the cost of the investment and more surgical time in case of computer navigation. Further costs for imaging and instruments in case of PSI are being generated [27, 28]. As some MRI-based PSI systems have reported issues with fitting accuracy, most TKA suppliers offer PSI together with a surgical plan to support implantation.

While the overall improvement of leg axis alignment using PSI may be small over conventional TKA, PSI has been shown to simplify surgery and safe operating time in several studies [27–29]. When PSI instrument were used with a kinematic alignment technique an improved knee flexion and better clinical outcomes were reported compared with mechanical alignment and conventional techniques [30]. This indicates that the underlying surgical principles are of essential relevance and that the employed instruments should be regarded as aids to pursue these principles for improved surgical outcomes.

Similarly, computer navigation-assisted TKA has successfully been employed for improving limb alignment and implant positioning while only very few studies could demonstrate that the improved radiographic outcomes necessarily have to be correlated with improved clinical outcomes [31]. Thus, navigation and PSI can be considered beneficial to reduce the surgeon error by facilitating accurate implant positioning and correction of leg alignment in TKA [24, 32]. Hence, this leads to improved outcomes provided that the surgical principles are correct.

Collectively, using the patient-specific iTotal™ CR G2 total knee replacement system enabled a proper fitting and positioning of the implant. Moreover, a decent restoration of the leg axis towards neutral alignment was achieved as planned. Nonetheless, we are aware that this study has several limitations. Firstly, we neither compare our operative technique to another surgical technique using conventional, computer navigated or PSI methods, nor can we compare the underlying surgical principle of anatomic reconstruction using patient-specific implants to other conventional TKA principles such as measured resection or gap balancing using of the shelf-implants. Moreover, this is a retrospective study focusing on the radiographic assessment and we did not investigate the clinical outcomes of patients in this study. Furthermore, we did not perform postoperative CT scans due to radiation protection. However, we believe that the accurate implant fit and alignment correction observed in our analysis using patient-specific instruments and implants demonstrate a promising pre-condition for potentially favourable outcomes. Nonetheless, this will have to be proven in further studies focusing on the clinical outcome comparing this technique with conventional TKA principles and techniques.

## Data Availability

The datasets used and analyzed during the current study are available from the corresponding author on reasonable request.

## Abbreviations

AP: antero-posterior
BKA: bi-compartmental knee arthroplasty
CAD: computer aided design
CAM: computer aided manufacturing
CT: computed-tomography
FDA: United States Food and Drug Administration
FMA: mechanical axis of the femur
HKA: hip-knee-ankle angle
MRI: magnetic resonance imaging
OA: osteoarthritis
PSI: patient specific implant
THA: total hip arthoplasty
TKA: total knee arthroplasty
TMA: mechanical axis of the tibia
UKA: unicondylar knee replacement
ZMA: zone of the mechanical axis
